# Malaria: Examining Persistence at the Margins of Endemicity over 15 Years in Saudi Arabia

**DOI:** 10.3390/medicina62020288

**Published:** 2026-02-01

**Authors:** Yasir Alruwaili

**Affiliations:** 1 Department of Clinical Laboratory Sciences, College of Applied Medical Sciences, Jouf University, Sakaka 72388, Saudi Arabia; ysalruwaili@ju.edu.sa; 2 Center for Health Research and Innovations, Deanship of Graduate Studies and Scientific Research, Jouf University, Sakaka 72388, Saudi Arabia

**Keywords:** malaria, Saudi Arabia, importation-driven transmission, regional heterogeneity, *Plasmodium falciparum*, *Plasmodium vivax*, spatiotemporal analysis, ARIMA forecasting

## Abstract

*Background and Objectives*: Malaria, a mosquito-borne parasitic disease caused by *Plasmodium* species, remains a public health concern in many tropical and subtropical regions. In Saudi Arabia, sustained control efforts have substantially reduced malaria transmission; however, regional heterogeneity and the growing contribution of imported infections continue to shape national malaria epidemiology. This study aimed to provide a comprehensive national assessment of malaria patterns in Saudi Arabia within a prevention-of-reintroduction framework. *Materials and Methods*: National malaria surveillance data reported by the Ministry of Health from 2010 to 2024 were analyzed to describe temporal trends in malaria burden and incidence, parasite species distribution, age structure, and seasonality across 20 health regions. Spatial heterogeneity was assessed using regional heatmaps and endemicity mapping. Transmission classification, parasite species, and age distribution were examined nationally and in greater detail for the Aseer and Jazan regions during 2021–2024. Future malaria trends through 2030 were projected using autoregressive integrated moving average (ARIMA) models. *Results*: Between 2010 and 2024, a total of 52,627 malaria cases were reported nationally, with marked interannual variability and an increase in incidence observed after 2020. Malaria burden was historically concentrated in seven endemic regions and subsequently became largely restricted to Aseer and Jazan. *Plasmodium falciparum* and *Plasmodium vivax*/*Plasmodium ovale* accounted for the majority of infections, with cases predominantly occurring among individuals aged ≥10 years. From 2021 onward, no indigenous malaria transmission was recorded; introduced cases were uncommon, and most infections were classified as imported. Forecasting analyses indicated stable national and regional malaria trends through 2030. *Conclusions*: Malaria in Saudi Arabia during 2010–2024 was characterized by pronounced regional heterogeneity and predominantly importation-driven dynamics. These findings underscore the importance of sustained surveillance, targeted interventions in high-burden regions, and continued vigilance to prevent the re-establishment of local transmission.

## 1. Introduction

Malaria is a mosquito-borne parasitic disease caused by *Plasmodium* species. It remains a major public health concern globally, despite substantial reductions in incidence and mortality over recent decades [1,2]. While many countries have achieved interruption of local malaria transmission, the persistence of malaria at low levels continues to be driven by ecological receptivity, human mobility, and sustained importation of infections [2]. In such settings, maintaining malaria-free or near-elimination status requires long-term surveillance and prevention of re-establishment strategies.

Across the Middle East, malaria transmission has become increasingly focal and geographically restricted. Most countries in the region are now malaria-free, with reported cases largely representing imported infections associated with travel and migrant populations [3,4]. Despite these achievements, residual transmission has historically persisted in limited areas, reflecting heterogeneous ecological conditions and population movement patterns [2]. Countries approaching elimination, therefore, remain vulnerable to reintroduction, particularly where competent vectors are present and cross-border or international travel is substantial [5,6,7]. Within this broader regional context, the Arabian Peninsula exemplifies this pattern, with vulnerability especially pronounced in Gulf Cooperation Council (GCC) countries that host large expatriate populations [4].

Saudi Arabia has implemented one of the earliest and most sustained malaria control programs in the region, beginning in the mid-20th century. These efforts resulted in the elimination of malaria from large parts of the country at different time points, including early interruption of transmission in the Eastern Province and prolonged absence of indigenous malaria in regions such as Al-Jouf in the north [3,4]. In the present epidemiological setting, Saudi Arabia is in an elimination-maintenance phase, with most regions classified as malaria-free and residual malaria risk largely confined to the south-western regions, where ecological suitability, historical endemicity, and population movement intersect [7,8,9,10].

A total of 17 *Anopheles* species have been recorded in Saudi Arabia, of which four (*Anopheles arabiensis*, *Anopheles sergentii*, *Anopheles stephensi*, and *Anopheles superpictus*) are recognized as competent malaria vectors. The geographic distribution and vectorial efficiency of these species have historically shaped malaria transmission patterns across the Kingdom, with *An. arabiensis* playing a dominant role in the south-western regions, while other vectors have been implicated in transmission in eastern, central, and northern areas [9].

Despite these achievements, malaria cases continue to be reported nationally in Saudi Arabia, predominantly as imported infections [11]. Recent studies have documented increases in malaria notifications in non-endemic regions such as the Eastern Province and Jeddah, largely attributable to imported cases rather than local transmission [12,13]. National and regional analyses further indicate that malaria in Saudi Arabia disproportionately affects older age groups and is dominated by *Plasmodium falciparum*, patterns typical of settings where sustained local transmission has been interrupted [4,14]. The near absence of malaria cases during COVID-19–related travel restrictions in Makkah further underscored the central role of travel-related importation in shaping contemporary malaria epidemiology [15].

Although several regional and time-limited studies have examined malaria trends in specific areas or periods in Saudi Arabia [7,16], comprehensive national analyses that integrate long-term temporal trends, spatial heterogeneity across all health regions, transmission classification, demographic structure, seasonality, and future projections remain limited. Recent forecasting studies have suggested stable malaria trends under current control conditions [17]; however, these assessments have generally been conducted independently of a unified, long-term national analysis covering both pre- and post-elimination phases. In addition, while Ministry of Health surveillance reports provide essential official statistics on malaria burden [18], they are not intended to support in-depth analytical synthesis of long-term spatiotemporal patterns, transmission dynamics, or forward-looking projections within a prevention-of-reintroduction framework.

Accordingly, this study analyzes Ministry of Health malaria surveillance data from 2010 to 2024 to provide a comprehensive national assessment of malaria epidemiology in Saudi Arabia. By integrating long-term temporal trends, regional heterogeneity, transmission dynamics, age and parasite species distributions, and time-series projections through 2030, this study adds analytical value beyond routine surveillance reporting and provides an evidence-based perspective to support sustained surveillance and prevention-of-reintroduction strategies.

## 2. Materials and Methods

### 2.1. Study Design and Data Sources

This retrospective observational study analyzed publicly available national malaria data reported by the Ministry of Health (MoH) of Saudi Arabia for the period 2010–2024. The dataset includes annual malaria case notifications aggregated at the national and health-region levels and comprises information on case counts, parasite species, transmission classification, age group, and month of diagnosis where available. Malaria surveillance data, population denominators used for incidence calculations, and regional classifications were obtained from publicly available Ministry of Health statistical yearbooks and official health statistics reports [18].

### 2.2. Study Area and Health Regions

**Figure 1:**
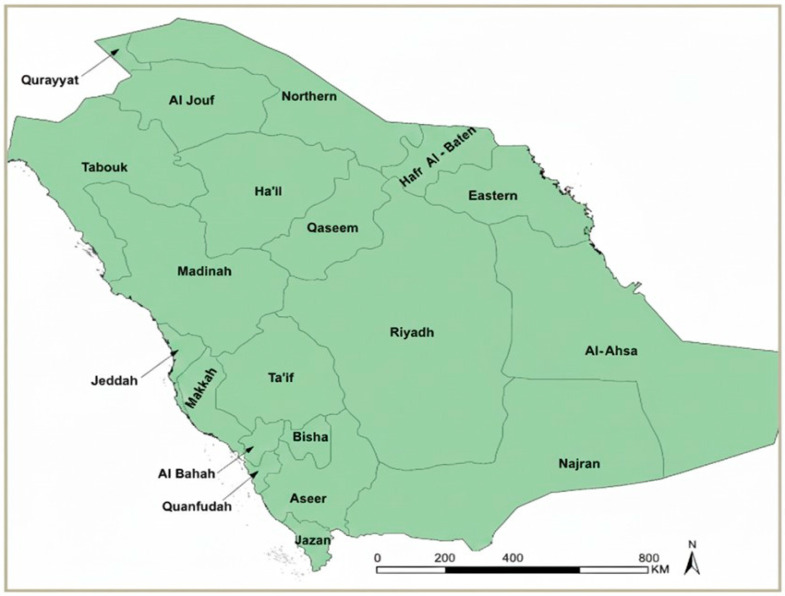
Health regions of Saudi Arabia. Map showing the 20 Ministry of Health (MoH) health regions of Saudi Arabia used for national and regional malaria analyses in this study. All regions are displayed uniformly to illustrate the spatial framework applied for malaria data aggregation and regional comparisons from 2010 to 2024. Administrative boundaries correspond to official MoH health regions and provide the geographic reference framework for all subsequent analyses.

Saudi Arabia is divided into 20 health regions within the 13 administrative regions, which served as the spatial units for regional analyses (Figure 1). All health regions were included in national trend analyses. For the purposes of this study, analyses were conducted across two distinct periods: 2010–2020 and 2021–2024. Historically, endemic regions were identified according to the Ministry of Health (MoH) classifications during 2010–2020, while updated MoH assessments for 2021–2024 indicated that malaria burden became largely confined to Aseer and Jazan. Accordingly, focused analyses for the later period were conducted for these two regions, which reported the highest malaria burden. Aseer and Jazan are characterized by mountainous and coastal ecologies, seasonal rainfall, and agricultural activity that support vector breeding; in addition, their proximity to international borders with historically endemic countries facilitates cross-border population movement, which remains an important driver of imported malaria risk in these regions [6,7,10,11]. Classification of malaria endemicity for both study periods was based exclusively on official Ministry of Health (MoH) definitions and endemicity classifications.

### 2.3. Case Definitions and Classification

Malaria cases were classified according to the Ministry of Health (MoH) surveillance definitions, whereby indigenous cases were defined as infections acquired locally with no epidemiological link to imported cases, introduced cases as locally acquired infections with clear epidemiological linkage to a confirmed imported case representing first-generation transmission, and imported cases as infections acquired outside the area in which the diagnosis was made. Parasite species were categorized as *Plasmodium falciparum* (malignant malaria), *Plasmodium vivax* and *Plasmodium ovale* (benign tertian malaria), *Plasmodium malariae* (benign quartan malaria), or mixed infections, defined as infections in which more than one *Plasmodium* species was identified in the same individual.

Age was grouped into three categories (<5 years, 5–9 years, and ≥10 years) in accordance with the structure of national malaria surveillance reporting used by the Ministry of Health over multiple years. Although more detailed age stratifications have been introduced in recent surveillance outputs, this categorization was retained to ensure consistency across the full study period and to allow a clear distinction between pediatric cases (<10 years) and predominantly travel- or importation-associated infections occurring among older individuals in elimination-phase settings. This pediatric-focused categorization is epidemiologically relevant, as malaria infections among children are more sensitive indicators of recent or local transmission, whereas cases among older age groups more commonly reflect exposure related to travel or population mobility.

### 2.4. Descriptive and Temporal Analyses

National malaria burden and incidence trends were summarized annually from 2010 to 2024. Incidence rates were calculated per 100,000 population using year-specific population denominators derived from official Ministry of Health (MoH) population statistics and national census estimates corresponding to each study year. Temporal patterns were examined at both annual and monthly scales to assess long-term trends and seasonality. Age- and parasite species–specific distributions were summarized using frequencies and proportions.

### 2.5. Spatial Analysis

Spatial heterogeneity in malaria burden was examined across the 20 health regions using regional aggregation. Heatmaps were generated to visualize annual variation in malaria case counts and incidence by region. Endemicity mapping was used to illustrate historically endemic regions during 2010–2020 and the subsequent geographic restriction of malaria burden during 2021–2024, based on Ministry of Health endemicity classifications.

### 2.6. Focused Regional Analysis (Aseer and Jazan, 2021–2024)

Focused analyses were conducted for Aseer and Jazan during 2021–2024 to characterize malaria transmission classification, parasite species distribution, and age-group profiles in regions reporting the highest malaria burden during the latter study period. Monthly case distributions were examined by transmission category (indigenous, introduced, and imported) to assess temporal dynamics and the relative contribution of each transmission type.

### 2.7. Time-Series Forecasting

Time-series forecasting of malaria cases was performed using autoregressive integrated moving average (ARIMA) models applied to annual total case counts. Separate models were fitted for the national, Jazan, and Aseer time series. Model selection was guided by inspection of autocorrelation and partial autocorrelation functions, as well as information criteria. Forecasts were generated for the period 2025–2030, with uncertainty expressed using 80% and 95% prediction intervals. Forecasts reflect historical total case patterns and do not model transmission categories separately.

### 2.8. Statistical Analysis

**Figure 2:**
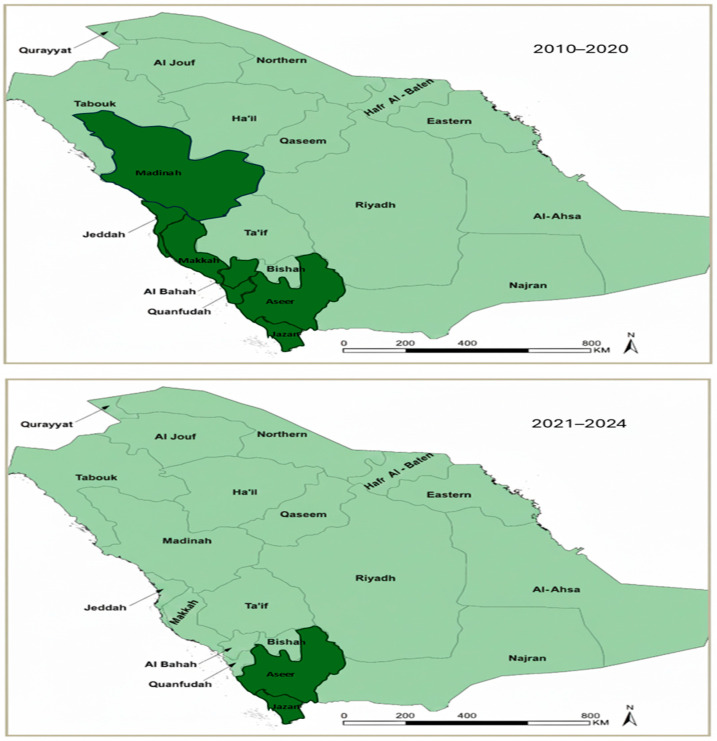
Spatial distribution of malaria endemicity in Saudi Arabia across two study periods. Spatial distribution of malaria endemicity across Saudi Arabia based on the Ministry of Health (MoH) classifications for two study periods. The upper panel shows health regions classified as malaria endemic during 2010–2020, including Makkah, Madinah, Jeddah, Al-Bahah, Qunfudah, Aseer, and Jazan. The lower panel shows the spatial distribution during 2021–2024, during which reported malaria cases were largely confined to Aseer and Jazan. Health regions are displayed uniformly to provide spatial reference. Dark-shaded regions indicate health regions classified as endemic during the specified period, while light-shaded regions represent non-endemic health regions.

All analyses were descriptive in nature. Results are presented as counts, proportions, and rates where appropriate. Missing regional data were not imputed and are reported as unavailable. Data cleaning, validation, and simple calculations were performed using Microsoft Excel (Microsoft Corporation, Redmond, WA, USA). Statistical analyses and data visualization were conducted using R software (R Foundation for Statistical Computing, version 4.5.2, Vienna, Austria) within the RStudio environment (Posit Software, version 2026.01.0+392, Boston, MA, USA). Additionally, a generative artificial intelligence tool was used solely to assist with the drawing of Figure 1 and Figure 2.

### 2.9. Ethical Considerations

This study used aggregated, publicly available secondary data with no individual-level identifiers. Ethical approval and informed consent were therefore not required.

## 3. Results

### 3.1. National Malaria Trends in Saudi Arabia (2010–2024)

Between 2010 and 2024, a total of 52,627 malaria cases were reported nationally in Saudi Arabia, with substantial interannual variability in both total case counts and incidence rates (Table 1). Annual malaria incidence increased from 7.15 per 100,000 population in 2010 to 19.95 per 100,000 in 2024. Case counts and incidence showed notable fluctuations over time, including a marked peak in 2016 and a sustained increase observed after 2020.

Throughout the study period, *Plasmodium falciparum* and *Plasmodium vivax*/*Plasmodium ovale* accounted for the majority of reported infections, together comprising more than 95% of cases in most years. The relative contribution of P. falciparum varied over time, ranging from 37.3% in 2012 to 88.3% in 2020, while *Plasmodium vivax*/*Plasmodium ovale* predominated in several earlier years, particularly between 2010 and 2013. In contrast, *Plasmodium malariae* and mixed-species infections were consistently uncommon, each accounting for a small proportion of annual cases across the study period.

As reported in Ministry of Health surveillance records, the majority of malaria cases during the study period were classified as imported.

### 3.2. Spatial Distribution and Regional Heterogeneity

Malaria burden was unevenly distributed across the 20 health regions of Saudi Arabia over the study period. During 2010–2020, reported malaria cases were concentrated in seven Ministry of Health classified endemic health regions, namely Makkah, Madinah, Jeddah, Al-Bahah, Qunfudah, Aseer, and Jazan, while the remaining regions consistently reported low or sporadic case numbers. From 2021 onward, the geographic distribution of reported malaria cases became more restricted, with the majority of cases originating from Aseer and Jazan and minimal case reporting from other health regions, reflecting the contraction of malaria endemicity during the elimination maintenance phase (Figure 2).

### 3.3. Regional Malaria Burden by Health Region (2010–2024)

**Figure 3:**
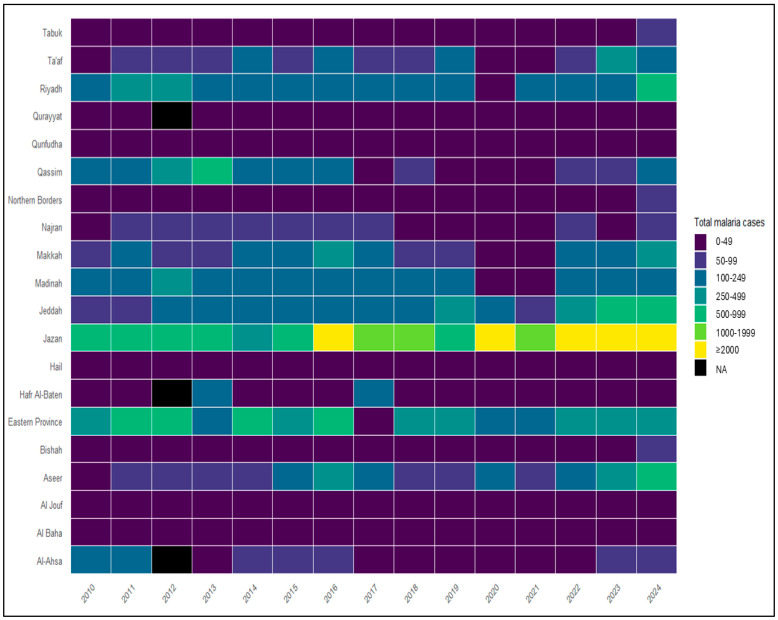
Heatmap of total malaria cases by health region and year in Saudi Arabia, 2010–2024. Heatmap displaying annual total malaria case counts by health region in Saudi Arabia from 2010 to 2024. Rows represent the 20 (MoH) health regions, and columns represent calendar years. Color intensity corresponds to the number of reported malaria cases per region and year, as indicated in the legend. Regional case data were not available for all health regions in 2013; missing values are shown in black and were not imputed.

**Figure 4:**
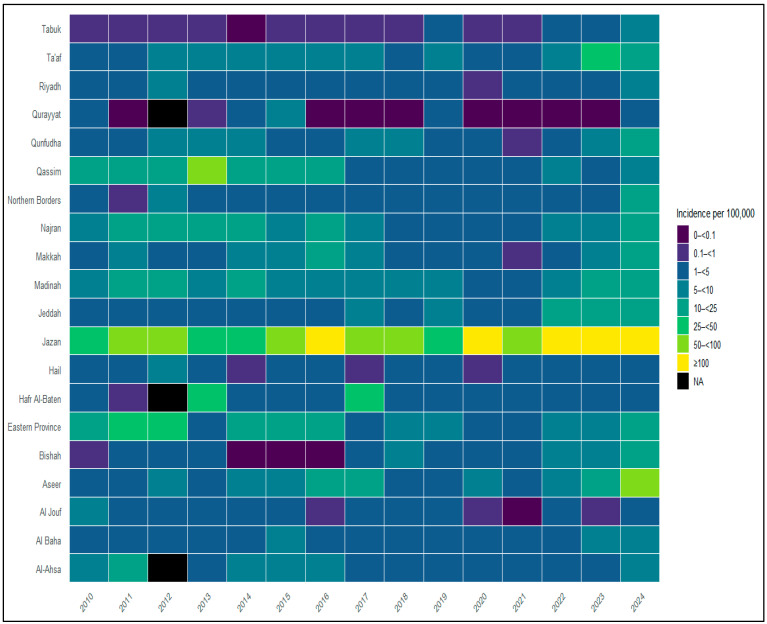
Heatmap of malaria incidence by health region and year in Saudi Arabia, 2010–2024. Heatmap displaying annual malaria incidence per 100,000 population by health region in Saudi Arabia from 2010 to 2024. Rows represent the 20 (MoH) health regions, and columns represent calendar years. Color categories correspond to incidence ranges as shown in the legend. Regional incidence data were not available for all health regions in 2013; missing values are shown as blank and were not imputed.

Regional analysis showed substantial variation in malaria case counts (Figure 3) and incidence (Figure 4) across the 20 health regions over the study period. Heatmap visualizations indicate that Aseer and Jazan consistently reported higher malaria case counts and incidence values across multiple years, particularly during the later part of the study period. Other historically endemic regions, including Makkah, Madinah, Jeddah, Al-Bahah, and Qunfudah, exhibited variable malaria burden, with higher case counts observed in some years during 2010–2020 and lower levels thereafter. Eastern Province, Ta’if, and Riyadh also showed periods of increased malaria case counts and incidence; however, all reported cases in these regions were classified as imported. From 2021 to 2024, all the reported malaria cases outside Aseer and Jazan were classified as imported, and only a few cases reported in Aseer and Jazan were classified as introduced. No indigenous malaria cases were reported nationally during this period.

### 3.4. Malaria Burden in Historically Endemic Regions (2010–2020)

To examine malaria patterns in greater detail, regional case data were analyzed separately for the two study periods, beginning with 2010–2020, during which malaria was reported across multiple MoH-classified endemic regions. During this first period, malaria cases were highly concentrated within a limited number of regions, with Jazan consistently contributing the largest share of cases, accounting for approximately 45–78% of endemic cases annually (Table 2). Other endemic regions, including Makkah, Jeddah, Madinah, and Aseer, contributed smaller and more variable proportions of cases across years. In contrast, Al-Bahah and Qunfudah consistently accounted for a minimal proportion of cases, together representing less than 3% of endemic cases in most years.

This period-specific analysis provides a detailed description of how malaria burden was distributed among endemic regions prior to the later geographic restriction observed after 2020, which is examined in subsequent sections.

### 3.5. Seasonal Patterns of Malaria in Endemic Regions (2010–2020)

**Figure 5:**
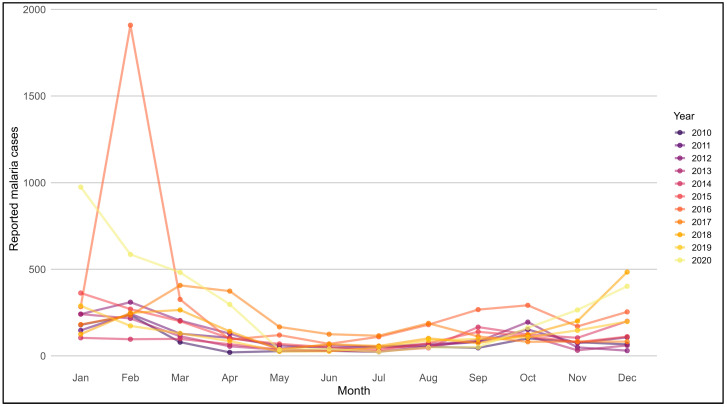
Monthly distribution of malaria cases in endemic regions of Saudi Arabia, 2010–2020. Line plot showing the monthly distribution of reported malaria cases in endemic regions of Saudi Arabia from 2010 to 2020. The x-axis represents calendar months (January–December), and the y-axis represents the number of reported malaria cases. Each line corresponds to a single calendar year. Endemic regions included in this analysis are Makkah, Jeddah, Madinah, Aseer, Jazan, Al-Bahah, and Qunfudah.

Monthly malaria case distributions in MoH-classified endemic regions showed recurrent seasonal patterns across the study period (Figure 5). In most years, malaria case counts began to increase during the first quarter of the year, with peak levels commonly observed between January and March, followed by a progressive decline during late spring and summer months. Case counts generally remained lower during mid- to late-year months, before rising again toward the beginning of the subsequent calendar year. While this seasonal pattern was observed consistently across endemic regions, the timing and magnitude of monthly peaks varied between years, with some years showing pronounced early-year peaks and others exhibiting more moderate monthly fluctuations.

### 3.6. Age Distribution of Malaria Cases in Endemic Regions (2010–2020)

**Figure 6:**
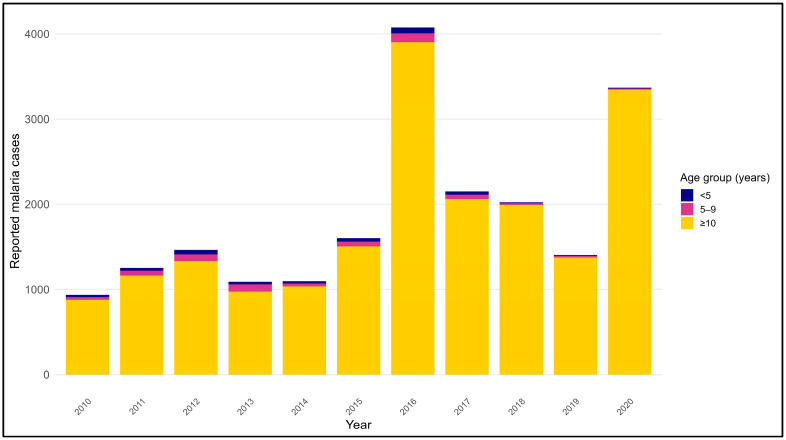
Age-group distribution of malaria cases in endemic regions of Saudi Arabia, 2010–2020. Stacked bar chart showing the annual distribution of reported malaria cases by age group in endemic regions of Saudi Arabia from 2010 to 2020. Age groups are categorized as <5 years, 5–9 years, and ≥10 years, and the y-axis represents the number of reported malaria cases. For each year, the total bar height reflects the overall annual case count, while the colored segments indicate the contribution of each age group. Endemic regions included in this analysis are Makkah, Jeddah, Madinah, Aseer, Jazan, Al-Bahah, and Qunfudah.

Across all study years, malaria cases in MoH-classified endemic regions were predominantly reported among individuals aged ≥10 years, who consistently accounted for the largest share of cases each year (Figure 6). In contrast, children under 10 years of age contributed a relatively small proportion of reported malaria cases throughout the study period. The distribution of cases among the three age groups (<5 years, 5–9 years, and ≥10 years) remained largely stable over time, with minimal interannual variation in the relative contribution of each age group. No marked shifts in age-group patterns were observed across the endemic regions during the 2010–2020 period.

### 3.7. Transmission Characteristics in Aseer and Jazan Regions (2021–2024)

During 2021–2024, no indigenous malaria transmission was recorded at the national level or in either the Aseer or Jazan regions (Table 3). Across all study years, the majority of reported malaria cases in both regions were classified as imported, while introduced cases accounted for a smaller proportion of infections. This transmission profile was consistently observed throughout the study period.

Marked differences in total malaria burden were observed between the two regions. Aseer reported relatively low annual case counts, increasing from 86 cases in 2021 to 869 cases in 2024, whereas Jazan recorded substantially higher case numbers, ranging from 1657 cases in 2021 to a peak of 3579 cases in 2023, followed by a modest decline in 2024 (Table 3). In all years, imported cases constituted the largest proportion of reported infections in both regions, exceeding 60% in Aseer and 90% in Jazan in most years.

Parasite species distribution showed similar patterns across regions and years. *Plasmodium falciparum* was the predominant species in both Aseer and Jazan, accounting for approximately 65–83% of reported cases annually. *Plasmodium vivax*/*Plasmodium ovale* represented a smaller but variable proportion of infections across years, while *Plasmodium malariae* and mixed-species infections were infrequently reported throughout the study period (Table 3).

Age-group distributions were consistent between regions. In all years, the majority of malaria cases occurred among individuals aged ≥10 years, accounting for more than 96% of reported cases annually in both Aseer and Jazan. Children aged <5 years and 5–9 years consistently represented a small proportion of cases, with minimal variation observed across years (Table 3).

**Figure 7:**
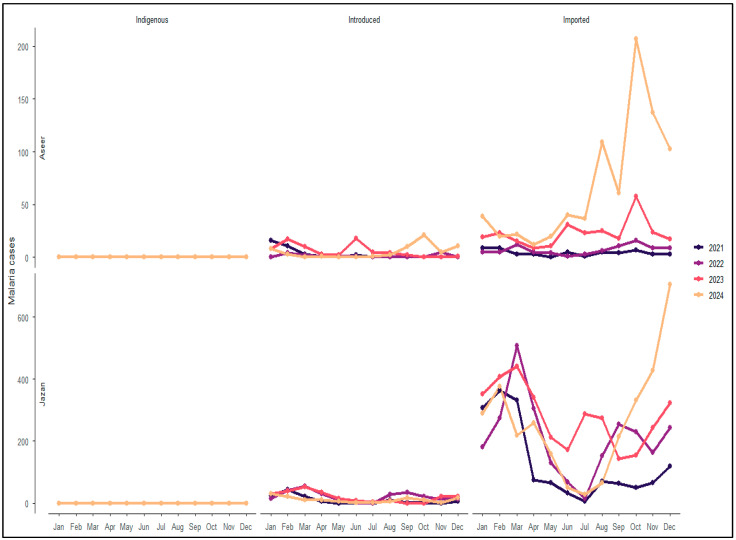
Monthly malaria cases by transmission category in Aseer and Jazan regions of Saudi Arabia, 2021–2024. Multi-panel line plots showing monthly malaria cases classified by transmission category (indigenous, introduced, and imported) in Aseer (top panels) and Jazan (bottom panels) regions of Saudi Arabia from 2021 to 2024. The x-axis represents calendar months (January–December), and the y-axis represents the number of reported malaria cases. Separate panels display each transmission category. Lines correspond to individual calendar years.

Monthly malaria case distributions further highlighted differences in the magnitude and temporal pattern of malaria burden between the two regions (Figure 7). Aseer consistently reported low monthly case counts throughout the year, with relatively limited variation between months. In contrast, Jazan recorded substantially higher monthly case numbers across all years, with clear month-to-month fluctuations and recurrent increases during the early months of the year, followed by lower case counts during late spring and summer. Despite these seasonal variations, higher monthly case numbers in Jazan were observed throughout the calendar year, while introduced cases represented a smaller proportion of reported infections in both regions.

### 3.8. Time-Series Forecasting of Malaria Cases (2025–2030)

**Figure 8:**
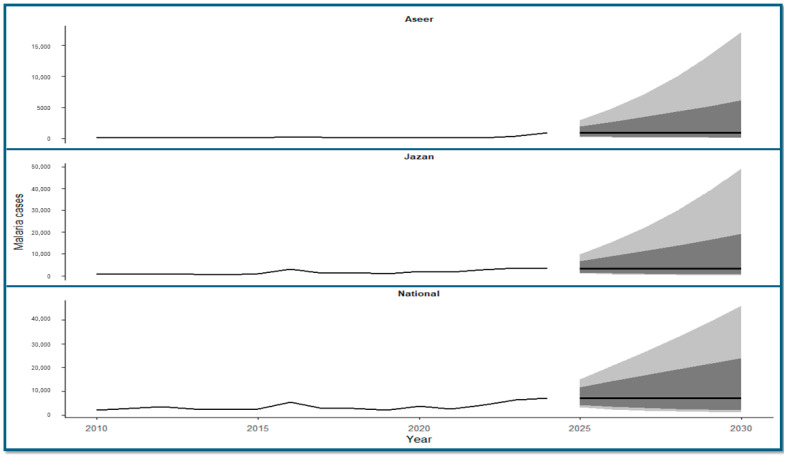
Forecasted malaria cases in Saudi Arabia at national and regional levels, 2025–2030. Multi-panel time-series plots showing observed malaria case counts (2010–2024) and forecasted malaria case counts (2025–2030) generated using autoregressive integrated moving average (ARIMA) models for Aseer (**top panel**), Jazan (**middle panel**), and Saudi Arabia nationally (**bottom panel**). The solid black line represents the annual malaria case counts, including observed cases up to 2024 and the ARIMA-predicted mean trend from 2025 to 2030. Forecast uncertainty is shown as shaded prediction intervals for 2025–2030, where dark shading indicates the 80% prediction interval and light shading indicates the 95% prediction interval. The x-axis represents calendar year, and the y-axis represents the number of reported malaria cases.

Time-series forecasting using autoregressive integrated moving average (ARIMA) models was used to project malaria case counts at national and regional levels through 2030 (Figure 8). At the national level, forecasted malaria cases remained within the range of values observed during recent years, with no abrupt deviations from historical patterns. In Jazan, projected case counts remained at levels comparable to those observed during the later years of the study period, with year-to-year variation reflected in the forecasted values. In Aseer, projections indicated lower case counts relative to Jazan, with modest increases observed over the forecast horizon. Across all forecasts, uncertainty increased over time, as shown by progressively widening prediction intervals.

## 4. Discussion

This national analysis provides a comprehensive assessment of malaria patterns in Saudi Arabia over a 15-year period, integrating temporal trends, spatial heterogeneity, transmission characteristics, and future projections. Overall, the findings indicate that malaria in Saudi Arabia is characterized by noticeable regional concentration and transmission dynamics driven largely by imported infections, reflecting the country’s advanced stage along the malaria elimination progress [2,3]. This pattern is consistent with regional and GCC-wide assessments showing that residual malaria burden in elimination settings is sustained primarily through importation rather than local transmission [5,6].

At the national level, malaria incidence exhibited marked interannual variability, including a notable peak in 2016 and renewed increases after 2020. Similar fluctuations have been observed in other countries approaching or maintaining malaria-free status and are often linked to changes in population movement, surveillance sensitivity, and exposure risk rather than re-establishment of sustained local transmission [2,19]. National surveillance data from Saudi Arabia likewise demonstrate that recent increases in reported cases are overwhelmingly attributable to imported malaria [11]. Within this context, the observed temporal variation and occasional inversion in the predominance of *Plasmodium* species are best interpreted as reflections of changing importation patterns rather than shifts in local transmission ecology. Variations in the relative contribution of *Plasmodium falciparum* and *Plasmodium vivax*/*Plasmodium ovale* likely correspond to differences in the geographic origin of imported cases, source-country epidemiology, and travel or migration flows over time. Notably, the regional data from Aseer and Jazan show that *Plasmodium falciparum* predominates among imported cases in recent years, supporting the interpretation that a substantial proportion of infections originates from regions where *P. falciparum* remains endemic.

Although individual-level nationality data were not available to directly link parasite species to specific countries of origin, temporal patterns in the national data provide additional contextual insight. In earlier years, when Saudi nationals constituted a larger proportion of reported cases, *Plasmodium vivax*/*Plasmodium ovale* predominated (Table 1). From approximately 2014 onward, a progressive shift toward *Plasmodium falciparum* dominance coincided with an increasing contribution of imported cases, suggesting a change in the epidemiological profile of malaria notifications rather than a resurgence of local transmission. Accordingly, the predominance of *Plasmodium falciparum* and *Plasmodium vivax*/*Plasmodium ovale* observed in this study aligns with previous reports from Saudi Arabia and neighboring regions, where imported malaria constitutes the majority of reported cases [4,10,13].

Spatial analyses revealed clear regional heterogeneity. During 2010–2020, malaria burden was concentrated in seven historically endemic regions, with Jazan consistently accounting for the largest share of cases. From 2021 onward, malaria burden became increasingly restricted to Aseer and Jazan, while most other regions reported minimal or no cases. This geographic contraction reflects the cumulative impact of sustained control measures and the interruption of transmission across much of the country [3,7,10]. Similar spatial concentration of imported malaria in southwestern and high-mobility regions has been documented in recent national analyses [11]. Nevertheless, the persistence of malaria in southwestern regions highlights the continued role of ecological receptivity, vector presence, and external exposure in shaping residual malaria risk [8,10,20].

Age-specific patterns further support the importation-driven nature of malaria in Saudi Arabia. The overwhelming predominance of cases among individuals aged ≥10 years contrasts sharply with high-transmission endemic settings, where young children bear the greatest burden of disease [2,21]. This age distribution is consistent with malaria acquired during travel or cross-border movement and mirrors findings from recent national and regional studies in Saudi Arabia [9,14]. Comparable adult-dominated age patterns have also been reported in imported malaria settings across the GCC [6]. Notably, the consistently low proportion of pediatric cases further supports the absence of recent local transmission, as malaria among children typically declines early during the elimination process and serves as a sensitive indicator of ongoing or recent transmission. Although individuals aged ≥10 years account for the majority of reported cases, this distribution reflects exposure and travel patterns rather than disease severity; children may still remain at higher risk for severe malaria outcomes despite representing a smaller proportion of cases in elimination-phase settings.

Seasonal analyses demonstrated recurrent early-year peaks in malaria cases during the historically endemic period, corresponding broadly with the rainy season in southwestern Saudi Arabia. Similar seasonal patterns have been described in Jazan and Aseer and are thought to reflect climatic conditions that enhance vector breeding, as well as seasonal mobility patterns that increase exposure risk [8,10,22]. Evidence from Makkah during COVID-19 travel restrictions further suggests that seasonality operates within a framework dominated by travel-related importation rather than sustained local transmission [15]. Importantly, the persistence of seasonality in the absence of indigenous transmission highlights the influence of imported infections entering receptive environments.

Focused analyses of Aseer and Jazan during 2021–2024 confirmed the absence of indigenous malaria transmission at both regional and national levels, with the majority of the cases classified as imported and a small proportion identified as introduced. These findings are consistent with earlier entomological, epidemiological, and modeling studies emphasizing the central role of importation in sustaining malaria risk in these regions [7,9,13]. Recent national surveillance similarly confirms that Jazan bears the highest imported malaria burden nationally [11]. The higher burden observed in Jazan compared with Aseer likely reflects differences in exposure intensity, population movement, and ecological suitability rather than intrinsic differences in control effectiveness.

Time-series forecasting using ARIMA models indicated stable malaria trends at both national and regional levels through 2030, with projected case counts remaining within ranges observed during the recent elimination-maintenance period. These findings align closely with recent machine-learning–based forecasts from Jazan, where Artificial Neural Network (ANN) models projected no significant increase in malaria incidence between 2020 and 2030 and identified associations with climatic factors [17]. The concordance between statistical and machine-learning approaches strengthens confidence that malaria risk in Saudi Arabia is unlikely to increase under current control conditions [2]. Importantly, the forecasted trends shown in Figure 8 should be interpreted as statistical extrapolations of historical surveillance data rather than deterministic predictions of future malaria burden. Although the upper bounds of the 80% and 95% prediction intervals widen over time, reflecting increasing uncertainty inherent in long-term forecasting, the central projected trajectories at both national and regional levels remain relatively stable and do not imply an anticipated resurgence of sustained local transmission, but rather reflect uncertainty under continued importation-driven conditions within a functioning surveillance and control framework.

From a public health and policy perspective, these findings support a prevention-of-reintroduction approach based on sustained, risk-based surveillance rather than uniform nationwide intensification, while maintaining routine malaria screening in settings such as blood donation services, travel clinics, and entry-point screening for newly arriving populations [2,4]. The results indicate that surveillance efforts should prioritize timely case detection, accurate transmission classification, and continued monitoring of epidemiological indicators most relevant to elimination-maintenance settings, including age distribution and parasite species profiles. Seasonal variation observed in historical data further suggests that preparedness and surveillance sensitivity may benefit from temporal alignment during higher-risk periods. At the clinical level, the continued predominance of *Plasmodium falciparum* among reported infections underscores the importance of sustained diagnostic capacity and prompt case management to mitigate the risk of severe disease and onward transmission. Collectively, these findings provide a practical evidence base to inform malaria surveillance priorities, prevention strategies, and policy planning during the elimination-maintenance phase in Saudi Arabia.

Several limitations should be acknowledged. Analyses relied on aggregated routine surveillance data, which may be affected by underreporting and temporal reporting variability, and did not permit assessment of individual-level or sex-specific risk factors. Incomplete regional data in selected years were not imputed, and forecasting models were based solely on historical case counts without explicit incorporation of environmental, entomological, or mobility covariates. Nonetheless, the use of long-term national surveillance data and complementary analytical approaches provides a robust and policy-relevant overview of malaria epidemiology in Saudi Arabia.

## 5. Conclusions

Malaria in Saudi Arabia over the period 2010–2024 was characterized by distinct regional heterogeneity and transmission dynamics dominated by imported infections. Sustained control efforts have successfully interrupted local transmission across most regions, with residual burden confined to historically endemic southwestern areas. Forecasts indicate stable malaria trends in the coming years; however, continued vigilance, targeted interventions in high-burden regions, and sustained monitoring remain essential to prevent re-establishment of local transmission and maintain current gains.

## Figures and Tables

**Table 1:** National malaria burden, incidence, and parasite species distribution in Saudi Arabia, 2010–2024.

Year	Total Cases	Incidence Per 100,000	*Plasmodium falciparum* (Malignant) *n* (%)	*Plasmodium vivax*/ *Plasmodium ovale* (Benign Tertiary) *n* (%)	*Plasmodium malariae* (Benign Quartan) *n* (%)	Mixed *n* (%)
**2010**	1941	7.15	883 (45.5)	1023 (52.7)	24 (1.2)	11 (0.6)
**2011**	2788	9.83	1045 (37.5)	1719 (61.7)	19 (0.7)	5 (0.2)
**2012**	3406	11.67	1270 (37.3)	2097 (61.6)	35 (1.0)	4 (0.1)
**2013**	2513	8.38	974 (38.8)	1527 (60.8)	6 (0.2)	6 (0.2)
**2014**	2305	7.49	1155 (50.1)	1144 (49.6)	6 (0.3)	0 (0.0)
**2015**	2620	8.31	1444 (55.1)	1164 (44.4)	10 (0.4)	2 (0.1)
**2016**	5382	16.96	3922 (72.9)	1420 (26.4)	40 (0.7)	0 (0.0)
**2017**	2715	8.34	1816 (66.9)	885 (32.6)	9 (0.3)	5 (0.2)
**2018**	2711	8.11	1898 (70.0)	802 (29.6)	1 (0.0)	10 (0.4)
**2019**	2152	6.29	1498 (69.6)	629 (29.2)	10 (0.5)	16 (0.7)
**2020**	3658	10.45	3231 (88.3)	419 (11.5)	0 (0.0)	8 (0.2)
**2021**	2616	7.67	2023 (77.3)	480 (18.3)	9 (0.3)	104 (4.0)
**2022**	4319	13.42	2079 (48.1)	1179 (27.3)	22 (0.5)	39 (0.9)
**2023**	6460	19.17	3769 (58.3)	2381 (36.8)	36 (0.6)	274 (4.2)
**2024**	7041	19.95	3798 (54.0)	2876 (40.9)	54 (0.8)	313 (4.4)

Percentages represent the proportion of total cases per year. The bold formatting in the table footer is intentional and is used to emphasize the main years of comparison, thereby improving clarity and readability.

**Table 2:** Annual total malaria cases reported from endemic regions of Saudi Arabia, 2010–2020.

Year	Makkah *n* (%)	Jeddah *n* (%)	Madinah *n* (%)	Aseer *n* (%)	Jazan *n* (%)	Al-Bahah *n* (%)	Qunfudah *n* (%)	Total
**2010**	92 (9.8)	66 (7.1)	145 (15.5)	46 (4.9)	561 (59.9)	13 (1.4)	13 (1.4)	936
**2011**	114 (9.1)	93 (7.4)	215 (17.2)	60 (4.8)	747 (59.6)	10 (0.8)	14 (1.1)	1253
**2012**	75 (5.1)	100 (6.8)	298 (20.4)	83 (5.7)	860 (58.8)	21 (1.4)	27 (1.8)	1464
**2013**	85 (7.8)	101 (9.3)	172 (15.8)	67 (6.1)	631 (57.9)	19 (1.7)	15 (1.4)	1090
**2014**	116 (10.6)	127 (11.6)	218 (19.9)	89 (8.1)	499 (45.5)	19 (1.7)	29 (2.6)	1097
**2015**	119 (7.4)	158 (9.9)	182 (11.4)	112 (7.0)	984 (61.4)	34 (2.1)	13 (0.8)	1602
**2016**	263 (6.5)	180 (4.4)	177 (4.3)	267 (6.6)	3157 (77.5)	18 (0.4)	13 (0.3)	4075
**2017**	207 (9.6)	238 (11.1)	201 (9.3)	193 (9.0)	1279 (59.5)	15 (0.7)	18 (0.8)	2151
**2018**	67 (3.3)	215 (10.6)	130 (6.4)	63 (3.1)	1516 (75.0)	12 (0.6)	19 (0.9)	2022
**2019**	87 (6.2)	257 (18.3)	133 (9.5)	90 (6.4)	818 (58.2)	9 (0.6)	11 (0.8)	1405
**2020**	36 (1.1)	147 (4.4)	28 (0.8)	113 (3.4)	2022 (60.0)	15 (0.4)	6 (0.2)	3367

Values are presented as number of cases, with percentages indicating each region’s contribution to the annual total of endemic cases. The bold formatting in the table footer is intentional and is used to emphasize the main years of comparison, thereby improving clarity and readability.

**Table 3:** Annual malaria characteristics in Aseer and Jazan regions of Saudi Arabia, 2021–2024.

Variable	2021		2022		2023		2024	
	Aseer	Jazan	Aseer	Jazan	Aseer	Jazan	Aseer	Jazan
**Total cases**	86	1657	100	2774	343	3579	869	3251
**Transmission category**								
**Indigenous** ***n* (%)**	0 (0.0)	0 (0.0)	0 (0.0)	0 (0.0)	0 (0.0)	0 (0.0)	0 (0.0)	0 (0.0)
**Introduced** ***n* (%)**	34 (39.5)	112 (6.8)	14 (14.0)	260 (9.4)	70 (20.4)	231 (6.5)	62 (7.1)	129 (4.0)
**Imported** ***n* (%)**	52 (60.5)	1545 (93.2)	86 (86.0)	2514 (90.6)	273 (79.6)	3348 (93.5)	807 (92.9)	3122 (96.0)
**Parasite species**								
***Plasmodium falciparum*** **(Malignant)** ***n* (%)**	66 (76.7)	1375 (83.0)	65 (65.0)	2284 (82.3)	242 (70.6)	2582 (72.1)	582 (67.0)	2099 (64.6)
***Plasmodium vivax*** **/** ***Plasmodium ovale*** **(Benign Tertiary)** ***n* (%)**	20 (23.3)	190 (11.5)	35 (35.0)	477 (17.2)	98 (28.6)	793 (22.2)	279 (32.1)	882 (27.1)
***Plasmodium malariae*** **(Benign Quartan)** ***n* (%)**	0 (0.0)	2 (0.1)	0 (0.0)	5 (0.2)	1 (0.3)	2 (0.1)	2 (0.2)	6 (0.2)
**Mixed** ***n* (%)**	0 (0.0)	90 (5.4)	0 (0.0)	8 (0.3)	2 (0.6)	202 (5.6)	5 (0.6)	264 (8.1)
**Age group (years)**								
**<5** ***n* (%)**	1 (1.2)	10 (0.6)	0 (0.0)	45 (1.6)	7 (2.0)	36 (1.0)	7 (0.8)	15 (0.5)
**5–9** ***n* (%)**	2 (2.3)	16 (1.0)	0 (0.0)	61 (2.2)	2 (0.6)	24 (0.7)	6 (0.7)	27 (0.8)
**≥10** ***n* (%)**	83 (96.5)	1631 (98.4)	100 (100.0)	2668 (96.2)	334 (97.4)	3519 (98.3)	856 (98.5)	3209 (98.7)

Values are presented as number of cases with percentages representing the proportion of total cases per region and year. The bold formatting in the table footer is intentional and is used to emphasize the main years of comparison, thereby improving clarity and readability

## Data Availability

The data supporting the findings of this study are publicly available from the Ministry of Health, Saudi Arabia, through statistical yearbooks and official health statistics reports (https://www.moh.gov.sa/en/Ministry/Statistics/book/Pages/default.aspx) (accessed on 1 January 2026). No new data were created in this study.

## Abbreviations

The following abbreviations are used in this manuscript:

ANN	Artificial Neural Network
ARIMA	Autoregressive Integrated Moving Average
COVID-19	Coronavirus Disease 2019
GCC	Gulf Cooperation Council
MoH	Ministry of Health
